# Antibacterial and Antifungal Activity of the Human Endometrial Fluid during the Natural Cycle

**DOI:** 10.1155/2021/8849664

**Published:** 2021-06-16

**Authors:** Marta Bregón-Villahoz, Maria-Dolores Moragues, Inés Arrieta-Aguirre, Mikel Azkargorta, Lucía Lainz, Miren Diez-Zapirain, Maria Iglesias, Maria-Begoña Prieto, Ana Matorras, Antonia Exposito, Felix Elortza, Roberto Matorras

**Affiliations:** ^1^Department of Nursing, Faculty of Medicine and Nursing, University of the Basque Country UPV/EHU, Barrio Sarriena s/n, 48940-Leioa, Spain; ^2^Proteomics Platform, CIC bioGUNE, CIBERehd, ProteoRed-ISCIII, Bizkaia Science and Technology Park, Derio, Spain; ^3^Human Reproduction Unit, Cruces University Hospital, University of the Basque Country, Barakaldo, Spain; ^4^Facultad de Medicina, Universidad Europea de Madrid, Villaviciosa de Odon, Madrid, Spain; ^5^IVIRMAI, IVI Bilbao, Spain

## Abstract

**Purpose:**

Some microbiota patterns have been associated with favorable IVF prognosis and others with pathological conditions. The endometrial fluid aspirate (EFA) contains antibacterial proteins that are enriched in implantative IVF cycles, but the antimicrobial effect of EFA has not been addressed. We aimed to evaluate the antimicrobial activity of the human endometrial fluid during the natural cycle.

**Methods:**

EFA was obtained through an embryo transfer catheter in 38 women, aged 18-40 years, with regular cycles attending to a fertility clinic. The antimicrobial activity of EFAs was tested against two strains of *Staphylococcus aureus*; one strain each of *Streptococcus agalactiae*, *Enterococcus faecalis*, *Escherichia coli*, and *Klebsiella pneumoniae*; and three yeasts (*Candida albicans*, *Candida glabrata*, and *Candida krusei*).

**Results:**

All samples exhibited antibacterial activity against *S. aureus*. In addition, 32.4% of EFAs were active against one of the other microorganisms assayed, 16.2% against two, and 5.4% against four of them. In contrast, none exhibited antibacterial activity against *E. coli* or *K. pneumoniae*. The antimicrobial activity differs considerably between EFA samples, and we failed to observe a cycle-related pattern.

**Conclusions:**

EFA presented two antimicrobial activity patterns: (a) one common to all the samples, exhibiting activity against *S. aureus* and lack of activity against *E. coli* and *K. pneumoniae*, and (b) an individualized pattern, showing activity against some of the other microorganisms tested. The intensity of antibacterial activity differs between EFA samples. Our data suggest that the uterine microbiota is controlled by means of endometrial fluid components.

## 1. Introduction

Embryo implantation is one of the most inefficient steps of assisted reproduction [1]. Implantation rates are usually <70% even if euploid embryos are transferred [2]. Given this, there is a growing interest in investigating the uterine role in implantation. Since the early works of Noyes et al. [3], it is well known that the endometrium undergoes histological changes which are pivotal for embryo implantation.

In recent years, a huge number of different microorganisms have been reported in different organs, such as the gut, skin, lungs [4], urinary bladder [5], and vagina [6]. Some reports have focused on endometrial microbiota. It has been reported that endometrial and vaginal microbiota can differ in structure and composition [7, 8]. Indeed, a microbiota pattern favorable for embryo implantation in in vitro fertilization (IVF) cycles has been described [8].

Moreover, the secretions of cultured human endometrial cells have shown antimicrobial activity [9–11], and antimicrobial peptides have been reported in them [12–14]. Nevertheless, the in vivo implication of this activity remains unknown.

We have previously shown that endometrial fluid can be easily aspirated [15] without impairing pregnancy rates, even if performed at the same time as embryo transfer [16]. Depending on the analytical approach and the applied criteria, somewhat more than 800 [17] or even over 2,200 proteins have been detected in the endometrial fluid aspirate (EFA) and some endogenous peptides of the EFA have shown antibacterial activity [18]. On the other hand, we have shown that in IVF the concentration of antibacterial peptides in the EFA is higher in cycles where implantation occurs than those where implantation fails [19].

However, actual antibacterial activity would presumably be the result of interaction between different antibacterial proteins with endogenous peptides or even with other molecules present in the endometrial fluid. Thus, we have sought to analyze the overall antibacterial activity of the EFA obtained during natural ovarian cycles.

## 2. Material and Methods

### 2.1. Population

The population under study consisted of 38 women of Caucasian ethnicity attending the Assisted Reproduction Unit from the Cruces University Hospital. The inclusion criteria were as follows: (i) age between 18 and 40 years; (ii) normal uterine ultrasound; (iii) cycle length of 28-30 days; (iv) previous normal cervical cytology; (v) absence of tubal conditions; (vi) no history of cervical infections, sexually transmitted diseases, pelvic inflammatory disease, endometriosis, or polycystic ovarian disease; vii) no previous miscarriages; (viii) 0-2 previous IVF cycles; (ix) no antibiotic or hormonal therapy in the last 4 months; (x) normal ovarian reserve; (xi) day of endometrial cycle 5-28; and (xii) body mass index (BMI) < 30 kg/m^2^.

Patients characteristics were as follows: mean age of 35.4 ± 3.8 years, duration of infertility of 2.4 ± 1.7 years, BMI of 26.9 ± 3.8 kg/m^2^, 94.4% having no previous children, 66.7% having no previous IVF cycles, and 22.2% smokers. In 42% of cases, the indication for IVF was the male factor.

The study was approved by our Institutional Ethical and Investigation Board (CEIC code 11/45).

### 2.2. Endometrial Fluid Collection

Patients were asked to participate in the study by donating an EFA sample during the mock embryo transfer, which is usually performed as a part of the pre-IVF protocol. Written informed consent was obtained.

A sterile disposable vaginal speculum was placed without employing vaginal lubricants. In cases where leucorrhea precluded access to the cervical external os, the discharge was gently removed with a sterile gauze. The EFA collection method has been recently reported [16, 19]. An “embryo transfer” catheter (Frydman catheter, Instrumentos Médicos Estériles, SA, Spain) was inserted through the cervical canal, in aseptic conditions, without it touching the vaginal walls or the ectocervix. Abdominal ultrasound guidance was used in order to facilitate the passing through the cervix [1] and to prevent the catheter from touching the uterine fundus. The sample was aspirated with gentle negative pressure applied with a 10 mL syringe connected to the catheter and 50-200 *μ*L of EFA were obtained [16, 19]. To prevent contamination with cervical fluid, aspiration was interrupted at the internal cervical os, and special care was taken to avoid traumatizing the uterine fundus or the cervix and contaminating the EFA sample with endometrial tissue or blood. Heavily blood-stained samples were discarded.

Since there is not a standardized technique to obtain EFA for antibacterial activity analysis, we used two methods. During the first part of the study, the samples (*n* = 20; EF-01 to EF-19 and EF-26) were expelled into standard cryogenic tubes, and the catheter tip was cut and placed into the same tube. In the second part of the study, after the expulsion of EFA, the inside of the catheter was rinsed with 1 mL of sterile saline solution. The samples were immediately frozen at −80°C until processed. Prior to be processed in the laboratory, the samples of the first group were filled up to 1 mL final volume with sterile saline and the catheter tip was removed after vortexing for 15 seconds. The first method carried the risk of dragging some germs from the cervical canal that could produce some antimicrobial substances. In the second case, the interior washing of the catheter would avoid this potential contamination of the sample.

EFA samples were not collected by both methods in the same patient because EFA is very scarce and there was a risk that the manipulations of obtaining the first sample would affect the quality of the second one.

### 2.3. Assessment of Microbial Growth from EFA Samples and Identification of Isolates

Ten *μ*L of each diluted sample, from both the “cut tip” sample group and the “flushed out catheter” group, was inoculated onto Columbia agar supplemented with 5% sheep blood (BD, USA) and incubated at 36 ± 1°C. Another 10 *μ*L aliquot of each sample was inoculated onto Man, Rogosa, and Sharpe agar (MRS; Sigma-Aldrich, USA) and incubated at 36 ± 1°C in a 5% CO_2_ atmosphere.

Bacterial DNA from the most frequent colony types isolated from samples was subjected to PCR amplification of the 16S rRNA gene using the bacterial universal primers 27F and 1522R as described by Kang et al. [20]. The amplicon sequences were compared with those stored in the NCBI database to identify the isolated bacteria.

### 2.4. Antimicrobial Activity of EFA Samples: Broth Microdilution Assay

EFA samples were gently homogenized in a rotating wheel for 30 min at 4°C and then centrifuged at 2,500 g for 15 minutes to eliminate the cellular debris.

The clarified EFA samples were assayed against three *Candida* strains (*Candida albicans* SC 5314, *Candida glabrata* ATCC 90030, and *Candida krusei* ATCC 6258) and six bacterial strains (*Escherichia coli* CECT 434, *Klebsiella pneumoniae* CECT 144, *Streptococcus agalactiae* CECT 183T, and *Enterococcus faecalis* CECT 481 and two *Staphylococcus aureus* strains—the CECT 435 reference strain and a methicillin-resistant clinical isolate). ATCC stands for American Type Culture Collection (Manassas, VA, USA) and CECT for Colección Española de Cultivos Tipo (Paterna, Valencia, Spain). *Candida* spp. were routinely grown for maintenance in Sabouraud agar (BD) at 36 ± 1°C for 24 h, while bacteria were grown in Nutrient Agar (Sigma-Aldrich) under the same conditions.

The antimicrobial activity of EFA was estimated in 96-well plates, following the guidelines of the Clinical and Laboratory Standards Institute (CLSI) protocols M-27-A3 for yeasts [21], and M07-A9 for bacteria [22], adapted to our conditions. Briefly, *Candida* strains were inoculated in 100 *μ*L of RPMI-MOPS, while bacteria were grown in Mueller-Hinton broth. Each well was supplemented with 25 *μ*L of the corresponding clarified EFA sample. Plates also included a positive growth control for each microorganism (with no EFA sample) and a negative culture medium control (no EFA sample and no microorganism), as well as growth controls for EFA samples with no added microorganisms. The plates were incubated at 36 ± 1°C, and growth inhibition with reference to the corresponding growth positive controls was assessed visually at 24 h (48 h for *S. aureus*) using the following scale: -, no inhibition; +, slight inhibition (less than 50% reduction of the cell pellet in the well); ++, moderate to high inhibition (≥50%); and +++, full growth inhibition. Growth was always blindly assessed by the same investigator (MB-V).

### 2.5. Statistical Analysis

The statistical association of categorical variables was analyzed with the chi-square or the Fisher's exact test, and *P* values < 0.05 were considered statistically significant (IBM® SPSS® Statistics v.24).

## 3. Results

### 3.1. Distribution of EFA Sampling over the Menstrual Cycle

Most EFA samples (28/38) were collected in the middle of the cycle between days 13 and 19 (EF-12, sampled on day 16, was excluded from the subsequent study of antimicrobial activity), while only five were collected between days 5 and 12 and another five in the late luteal phase, between days 24 and 28 (Table 1).

Twenty samples included the tip of the catheter, whereas the remaining 18 endometrial fluids were flushed out from the catheter. In both cases, EFA samples were made up to 1 mL final volume with sterile saline before being processed in the laboratory.

### 3.2. Microbial Growth of EFA Samples

Around 50% of the EFA samples showed no microbial growth on Columbia agar (17/38) or MRS agar (21/38) (Table 2). The proportion of cases with microbiological growth was significantly higher for EFA samples stored with the catheter tip compared with those which were flushed out (80% vs. 27.8% on Columbia agar (*χ*^2^ = 8.44; *p* < 0.05) and 60% vs. 27.8% on MRS agar (*χ*^2^ = 3.98; *p* < 0.05)). The analysis of the 16S rRNA gene sequences of colonies grown from 15 EFA samples showed that the most abundant and frequently isolated microbes were Gram-positive rod-shaped bacteria (*Lactobacillus* spp., mostly *Lactobacillus gasseri*), followed by colonies of Gram-positive coagulase-negative staphylococci (*Staphylococcus epidermidis*) in five samples (EF-04, 06, 10, 12, and 26). In contrast, other colonies from EF-02, 10, and 30 that only grew on Columbia agar were identified as *Actinomyces urogenitalis* and *Corynebacterium* spp.

The frequency of positive microbial growth was 2-3 times higher for samples that had retained the catheter tip than for those that only contained the internal washing fluid of the catheter. In addition, the inoculum from three out of the five fluids with no catheter but positive growth on MRS agar only yielded 1-2 colonies. In contrast, one of the samples that included the catheter (EF-12, collected on day 16 of the menstrual cycle) yielded uncountable colonies; since excessive microorganisms interfered with the antimicrobial activity assays, this sample was discarded for further analyses.

### 3.3. Antimicrobial Activity of EFA Samples

**Figure 1:**
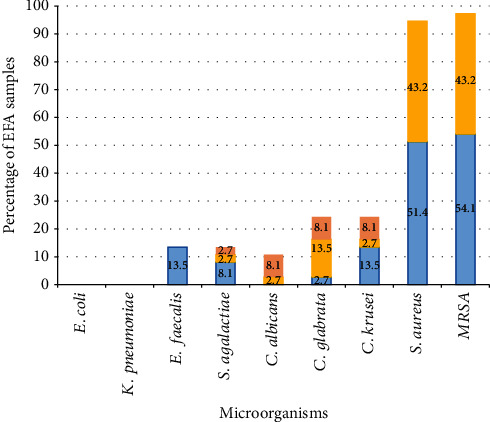
Percentage of EFA samples with antimicrobial activity against different microorganisms. Color code refers to the intensity of inhibition as described in Material and Methods (blue: +; yellow: ++; salmon: +++).

The data of antimicrobial activity displayed in Table 1 are summarized in Figure 1. Almost all the EFA samples studied (34/37; 91.9%) inhibited the growth of the two *S. aureus* strains tested, while the remaining three samples only inhibited one of them; none of the EFA samples exhibited full growth inhibition of *S. aureus* (Table 1). In addition to inhibiting *S. aureus*, 32.4% (12/37) of samples were active against only one of the other microorganisms tested, 16.2% (6/37) against two microorganisms, and 5.4% (2/37) against four microorganisms (Table 1).

Overall, 40.5% (15/37) of EFA samples reduced the growth of at least one of the three species of *Candida*, and five of them achieved full growth inhibition against them. Some other EFAs were active against *S. agalactiae* (13.5%) or *E. faecalis* (13.5%). In contrast, none of the diluted EFAs showed activity against *E. coli* or *K. pneumoniae* (Table 1; Figure 1).

Regarding the day of EFA collection during the menstrual cycle, all samples exhibited a certain degree of activity against *S. aureus* independently of the day of collection (Table 1). Four out of five samples that reduced the growth of *E. faecalis* were obtained in the central period of the cycle (days 14-17) and the fifth one on day 25 (Table 1). While *C. glabrata* and/or *C. krusei* were inhibited by several fluids collected at different points during the cycle (Table 1), *C. albicans* was inhibited only by four EFA samples from days 13 and 14; thus, the activity against *C. albicans* was statistically associated (*p* < 0.05) with EFA samples from the first part of the cycle (days 1-14) compared to the second part (days 14-28). Three EFA samples—EF04, EF06, and EF26—were active against two species of *Candida* (*C. glabrata* and *C. krusei*) and two—EF01 and EF27—against all three species tested (*C. albicans*, *C. glabrata*, and *C. krusei*).

Although care was taken to avoid epithelial bleeding during the sample collection, antibacterial activity against *S. agalactiae* was only registered in five out of 20 samples that contained traces of blood (EF-01, 05, 11, 14, and 25; Table 1).

As for the antimicrobial activity of EFA depending on the presence of culturable microorganisms in the samples, we found no differences in anti-*S. aureus* activity: all samples inhibited the growth of at least one of the two strains tested (Table 1). However, antimicrobial activity against the rest of microorganisms tested was somewhat more frequent in cases where the microbial culture of EFA on MRS and/or Columbia agar was positive (15/23; 65.22%) than those with no culturable bacteria (5/14; 35.71%) (*χ*^2^ = 3.05; *p* = 0.08) (Table 1). In regard to the collection method and storage, samples that included the catheter tip were more frequently associated with activity against microorganisms other than *S. aureus* (14/19; 73.68%) than those that contained only the flushing fluid from inside the catheter (6/18; 33.33%), and this association was statistically significant (*χ*^2^ = 6.06; *p* < 0.05).

## 4. Discussion

Microbial populations of varied composition have been described in association with different anatomical locations of the human body [23]. The interaction between the host and microbiota has been shown to play an essential role in many aspects of human physiology [24–26].

Much less is known about the uterine side of implantation than the embryonic side. The challenges of investigating the uterine side of implantation include endometrial cyclic changes, intercycle variability, and the influence of ovarian stimulation on endometrial changes, as well as the potential adverse effect of endometrial biopsy on implantation, if performed close to the time of embryo transfer [16]. In this context, we have focused our study on EFA, which can be obtained at the same time as embryo transfer, without impairing implantation rates [16].

Moreno et al. [8] identified a favorable pattern in the endometrial microbiota for the successful implantation of the embryo in IVF, characterized by a high proportion of *Lactobacillus*. In a recent study focusing on the protein composition of EFA obtained at the same time as the embryo transfer, we showed that EFA from IVF cycles where implantation took place (“implantative” cycles) was richer in antibacterial proteins than EFA from “nonimplantative” cycles [19]. This is in agreement with very recent data from an endogenous peptidomics-focused mass spectrometry analysis of EFA describing the presence of a number of peptides with potential antibacterial activity in this fluid [18]. In line with this, selected in silico predicted antibacterial peptides were synthesized and tested in vitro for antimicrobial activity. Preliminary results showed that, indeed, some of these peptides present antibacterial activity [18]. In the same way, it has recently been suggested that the innate immune system senses pathogen-associated molecular patterns and this could induce the release of antimicrobial peptides into the uterine cavity [27].

In the present study, we have evaluated the antimicrobial activity of EFA, which contains many different antibacterial proteins and peptides, whose synergisms or even antagonisms are still unknown. Indeed, some microorganisms present in EFA could also play an antibacterial role. For instance, the *Lactobacillus* genus produces lactic acid and short-chain fatty acids, acidifying the environment to pH ≤4.5 in the vagina and prohibiting the growth of other pathogenic or dysbiotic bacteria in healthy women [28, 29]. However, concerning the endometrium, no correlation has been observed between the pH value and the endometrial microbiota [8].

The possibility of sample contamination is a significant hurdle to investigate uterine microbiota [30]. There is no standardized methodology for the study of human uterine microbiota [30]. In most of the studies, the samples were collected through the cervix [30]. Some authors used endometrial biopsies [31], others used transcervical aspiration through an embryo transfer catheter [8, 32–34] and then emptied the content (without flushing out the catheter), others used a double lumen catheter, without aspiration, and analyzed the distal portion of the transfer catheter [7, 35], and some combined a double lumen ET catheter with lavage and endometrial biopsy [36]. Aspiration of EF under aseptic conditions has been shown to be a safe and effective method to evaluate the endometrial microbiota [8, 37]. We have undertaken this work in order to evaluate the potential antimicrobial activity of the endometrial fluid of 38 women in reproductive age. The EFA samples were collected through aspiration with an embryo transfer catheter passed through the cervical os, taking precautions to avoid contamination with the cervix microbiota. Two different collection methods were used: (i) emptying the catheter content and storing the sample with the catheter tip and (ii) flushing the inside of the catheter with saline solution. In our work, approximately half of the EFA samples contained culturable microorganisms, in agreement with data stating that the endometrial cavity is not sterile in most healthy women [38].

Twice as many positive cultures were obtained from samples stored with the catheter tip compared to samples without it. Colonies of *Staphylococcus epidermidis* were only identified in those samples stored with the catheter. It could be speculated that the external surface of the catheter retained a number of microorganisms from the cervical canal, and hence, for microbiological analyses, it seems advisable to collect only the internal fluid of the catheter. Nevertheless, the viable bacteria identified in both sample types consisted mainly of *Lactobacillus* spp. and to a much lesser extent *Actinomyces urogenitalis* and *Corynebacterium* spp. *Lactobacillus* spp. were the most frequently isolated bacteria from the EFA samples, in agreement with previous reports of Wee et al. [39] and Moreno and Simon [37]. Although significant differences have been reported in vaginal and uterine microbiota [8, 33, 34, 40], as far as we know, cervical and uterine microbiota have not been compared. Selman et al. studied endocervical and ectocervical samples as well as the internal tip of the catheter, but they did not aspirate samples and the results for each localization were not compared [35].

There is also a controversy about the method of analysis of the uterine microbiome. Regarding the culture of viable microbes, less than 1% of the bacteria present in a sample grow and form colonies; furthermore, some human samples may contain a limited amount of microorganisms [41]. On the other hand, the next-generation sequencing (NGS) analysis of EFA samples containing a small number of microorganisms may be distorted by abundant contaminating vaginal microbiota [30]. Moreover, NGS readings do not differentiate between live and dead microorganisms, and consequently, the uterine microbial population may be overestimated.

The uterine microbiota have been reported to fluctuate with menstrual cycle timing [42], ovarian stimulation [43], ethnic group [44], certain pathological conditions [42], and IVF prognosis [37]. In our study, performed in natural cycles of Caucasian women in reproductive age, we have not been able to establish a clear association between the viable microbiota of EFA samples and the time of sampling during the menstrual cycle. However, we acknowledge that only a small number of the samples studied were outside the central part of the cycle.

Regarding the antimicrobial activity, all the EFA samples in the present work inhibited the in vitro growth of *S. aureus* to some extent, and there were no significant differences between culturable and nonculturable samples, or even between samples collected at different times over the menstrual cycle. A number of potential antibacterial peptides have been reported from human endometrial tissue cultures [12–14]. Our results are consistent with those obtained with cultures of uterine epithelial cells [45], showing the production of antibacterial factors that effectively killed *S. aureus*, and correlation with secretory leukocyte protease inhibitor concentrations. Nevertheless, in the same study on uterine epithelial cell culture [45], antibacterial activity against *E. coli* was also observed, a type of activity that was not observed in any patients from our study. We were also unable to find any activity against *K. pneumoniae* in any of our patients' EFA samples. On the other hand, more than half of the EFA samples of the studied women (54.1%) exhibited antimicrobial activity against other microorganisms including *C. albicans*, *C. glabrata*, *C. krusei*, *S. agalactiae*, and/or *E. faecalis*. Moreover, our findings regarding *C. albicans* are in agreement with those of Wira et al. [46], who reported that secretions of cultures from the upper female reproductive tract cells inhibit yeast and hyphal forms of *C. albicans*.

It has to be stressed that there was a remarkable heterogeneity in antimicrobial activity patterns: (i) the intensity of the antimicrobial activity differs notably between the different specimens, and (ii) apart from *S. aureus* which was always inhibited, the proportion of EFA inhibiting some of the remaining microorganisms assayed ranged from 10.8 to 24.3%. Furthermore, in addition to *S. aureus*, 32.4% of EFA samples were active against only one of the remaining microorganisms assayed, 16.2% against two microorganisms, and only 5.4% against four microorganisms. On the other hand, it must be highlighted that a number of microorganisms, especially anaerobic bacteria, were not evaluated in our study.

Concerning the source of antibacterial compounds, in addition to endometrial tissue, as suggested by tissue culture studies, one cannot rule out an associated effect coming from the microbiota of the endometrial cavity. In agreement with this, apart from the anti-*S. aureus* activity which was present in every sample, we found that antimicrobial activity against the other tested microorganisms was more frequent in the samples where microbiological cultures were positive. Wang et al. [47] attributed antimicrobial activity to *Lactobacillus* spp. that produces lactic acid and hydrogen peroxide, among other compounds, which can inhibit the growth of potentially pathogenic bacteria, as well as the filamentation of *C. albicans*. Nonetheless, the anaerobic environment of the endometrium does not facilitate hydrogen peroxide production by *Lactobacillus*. Indeed, we have documented anti-*Candida* activity in more than one-third of the EFA samples, even in cases when no growth of *Lactobacillus* on MRS medium was recorded. Regarding the method of collection and storage, the samples containing only the fluid flushed out from the catheter were associated with the least frequently positive cultures and antimicrobial activity. Although our sample numbers were limited and we were unable to assess a range of confounding factors, the latter method is probably the one that can provide the most reliable information on the uterine microbiota and its antimicrobial activity.

In this study, we have shown that EFA samples from women of reproductive age exhibit antimicrobial activity against some microorganisms that could impair IVF results. All the EFA samples tested in vitro reduced the growth of *S. aureus*, and many of them inhibited *Candida* spp., *E. faecalis*, and/or *S. agalactiae* as well, but not *E. coli* or *K. pneumoniae*. To the best of our knowledge, this is the first report on the direct antimicrobial activity of endometrial fluid aspirate samples in humans.

Further studies are warranted to characterize the endometrial fluid of women undergoing IVF procedures in order to assess whether there is a relationship between the antimicrobial activity of raw EFA and implantation success.

## Figures and Tables

**Table 1:** Time distribution, sample characteristics and antimicrobial activity of EFA samples.

Cycle day	Sample			Activity against microorganisms^a^								
	Identification	Catheter tip ^b^	Microbial growth	*E. coli*	*K. pneumoniae*	*E. faecalis*	*S. agalactiae*	*S. aureus*	MRSA	*C. albicans*	*C. glabrata*	*C. krusei*
5	EF-05	Yes	Yes	-	-	-	+	-	+	-	-	+

10	EF-07	Yes	Yes	-	-	-	-	++	++	-	-	-
	EF-22	No	No	-	-	-	-	+	+	-	-	-

11	EF-24	No	Yes	-	-	-	-	+	-	-	-	+++

12	EF-23	No	No	-	-	-	-	+	++	-	-	-

13	EF-01	Yes	Yes	-	-	-	++	++	++	+++	+++	+++
	EF-02	Yes	Yes	-	-	-	-	+	++	-	-	-
	EF-10	Yes	Yes	-	-	-	-	++	+	-	++	-
	EF-28	No	No	-	-	-	-	-	+	++	-	-
	EF-29	No	No	-	-	-	-	++	+	-	-	-

14	EF-15	Yes	Yes	-	-	-	-	+	+	-	-	-
	EF-20	No	No	-	-	-	-	++	+	-	-	-
	EF-21	No	Yes	-	-	-	-	+	++	+++	-	-
	EF-25	No	No	-	-	-	+	++	++	-	-	-
	EF-27	No	No	-	-	+	-	+	++	+++	+++	+++

15	EF-04	Yes	Yes	-	-	-	-	+	++	-	++	+
	EF-19	Yes	Yes	-	-	-	-	+	+	-	++	-
	EF-30	No	Yes	-	-	-	-	++	++	-	-	-
	EF-37	No	No	-	-	-	-	++	+	-	-	-

16	EF-03	Yes	Yes	-	-	-	-	+	++	-	-	+
	EF-13	Yes	Yes	-	-	+	-	+	+	-	-	-
	EF-16	Yes	No	-	-	+	-	++	++	-	-	-
	EF-26	Yes	Yes	-	-	-	-	+	++	-	+	++
	EF-31	No	Yes	-	-	-	-	++	+	-	-	-
	EF-35	No	Yes	-	-	-	-	++	++	-	-	-
	EF-36	No	Yes	-	-	-	-	++	++	-	-	-

17	EF-33	No	No	-	-	+	-	++	+	-	-	-
	EF-34	No	No	-	-	-	-	+	+	-	-	-

18	EF-06	Yes	Yes	-	-	-	-	+	+	-	+++	+
	EF-14	Yes	Yes	-	-	-	+++	+	+	-	-	-

19	EF-18	Yes	Yes	-	-	-	-	+	+	-	-	-
	EF-32	No	No	-	-	-	-	+	+	-	-	-

24	EF-17	Yes	No	-	-	-	-	+	+	-	-	-

25	EF-11	Yes	Yes	-	-	-	+	++	++	-	++	-

26	EF-08	Yes	Yes	-	-	-	-	+	++	-	-	+

27	EF-09	Yes	Yes	-	-	+	-	++	+	-	++	-

28	EF-38	No	No	-	-	-	-	++	+	-	-	-

^a^Antimicrobial activity with reference to the cell pellet of the positive growth control well. -: no inhibition; +: slight inhibition (<50% reduction); ++: moderate to high inhibition (≥50%); +++: full growth inhibition. ^b^Catheter: “yes” stands for samples stored with the catheter tip prior to processing; “no” stands for samples flushed out from the catheter before being stored. Microorganisms: *Escherichia coli*, *Klebsiella pneumoniae*, *Enterococcus faecalis*, *Streptococcus agalactiae*, *Staphylococcus aureus*, MRSA (*Methicillin-resistant S. aureus*), *Candida albicans*, *Candida glabrata*, and *Candida krusei*.

**Table 2:** Microbial growth of endometrial fluid aspirate (EFA) samples from 38 women on Columbia agar and MRS agar. The ratio of EFA samples displaying microbial growth in both media was significantly higher for those stored with the catheter tip (^∗^*χ*^2^ = 8.44, *p* < 0.05; ^∗∗^*χ*^2^ = 3.98, *p* < 0.05) compared to EFA samples flushed out from the catheter.

		Microbial growth of EFA samples on							
		Columbia agar				MRS agar			
		Positive		Negative		Positive		Negative	
EFA samples	*N*	*N*	%	*N*	%	*N*	%	*N*	%
With catheter tip	20	16	80^∗^	4	20	12	60^∗∗^	8	40
Flushed out from catheter	18	5	27.8	13	72.2	5	27.8	13	72.2
Total	38	21	55.3	17	44.7	17	44.7	21	55.3

## Data Availability

The data used to support the findings of this study are available from the corresponding author upon request.
